# A *Drosophila* model for developmental nicotine exposure

**DOI:** 10.1371/journal.pone.0177710

**Published:** 2017-05-12

**Authors:** Norma Andrea Velazquez-Ulloa

**Affiliations:** Biology Department, Lewis & Clark College, Portland, Oregon, United States of America; Biomedical Sciences Research Center Alexander Fleming, GREECE

## Abstract

Despite the known health risks of tobacco smoking, many people including pregnant women continue smoking. The effects of developmental nicotine exposure are known, but the underlying mechanisms are not well understood. *Drosophila melanogaster* is a model organism that can be used for uncovering genetic and molecular mechanisms for drugs of abuse. Here I show that *Drosophila* can be a model to elucidate the mechanisms for nicotine’s effects on a developing organism. *Drosophila* reared on nicotine food display developmental and behavioral effects similar to those in mammals including decreased survival and weight, increased developmental time, and decreased sensitivity to acute nicotine and ethanol. The *Drosophila* nicotinic acetylcholine receptor subunit alpha 7 (D**α**7) mediates some of these effects. A novel role for D**α**7 on ethanol sedation in *Drosophil*a is also shown. Future research taking advantage of the genetic and molecular tools for *Drosophila* will allow additional discovery of the mechanisms behind the effects of nicotine during development.

## Introduction

Tobacco addiction is a worldwide public health issue, accounting for nearly 6 million deaths a year [1,2]. According to the US National Survey of Drug Use and Health 15% of pregnant women use tobacco [3]. Tobacco addiction is a complex disease with social and biological factors. Importantly, twin studies estimate that about 50% of the differences in smoking habits and smoking cessation success can be explained by genetic factors [4,5].

Nicotine is the chemical in tobacco associated with its addictive effects [6,7,8]. Nicotine activates nicotinic acetylcholine receptors (nAChRs) in the brain. These receptors are normally activated by the endogenous ligand, acetylcholine, and have roles in learning and memory, psychomotor behaviors and reward [9]. Nicotinic receptor signaling is also important for normal development of the nervous system including roles in synapse formation, neuronal growth, neuronal differentiation, and the regulation of the GABA switch from an excitatory role early in development to its mature role as inhibitory neurotransmitter [10–12]. Therefore, exposure to nicotine during development has the potential of affecting several aspects of normal brain development by activating nAChRs in a non-physiological manner.

Known outcomes of developmental exposure to nicotine in humans and rodents are increased mortality, and low birth weight [13,14]. Behavioral effects in humans include increased incidence of nicotine addiction by adolescence, increased ethanol abuse and increased susceptibility for attention deficit hyperactivity disorder [15–17]. In rodents, behavioral effects of prenatal nicotine exposure include hyperactivity and increased nicotine self-administration [13,18,19]. A known outcome of prenatal nicotine exposure in mammals is an increase in the number of nicotinic binding sites [18,20,21]. However, the molecular mechanisms for this increase remain an area of active research.

Despite knowledge of the molecular targets and brain regions where nicotine acts, the neuroadaptations underlying the effects of nicotine exposure during brain development are not fully understood. The genetic, molecular and cellular mechanisms for prenatal nicotine thus warrant further investigation.

The fruit fly *Drosophila melanogaster* has been successfully established as model organism to study the effects of alcohol exposure in adult and developing flies [22–24]. A recent study also used *Drosophila melanogaster* as model organism to identify novel genes involved in adult nicotine sensitivity [25]. These studies have demonstrated that *Drosophila melanogaster* is a powerful model organism to identify novel genetic factors and molecular mechanisms that regulate responses to drugs of abuse.

There are ten genes encoding nAChR subunits in *Drosophila* [26,27]. The gene encoding the alpha7 subunit in Drosophila (*D***α***7*) is among the ones with the highest homology to vertebrate subunits [28]. Fayyazuddin et al. [29] showed that *D***α***7* is required for the giant-fiber mediated escape response. They characterized the expression pattern for D**α**7 protein and found that in addition to expression at the dendrites of the giant fiber, D**α**7 was also expressed in the neuropil of a wide range of regions of the adult brain, including the antennal lobes, the subesophageal ganglion and the calyces of the mushroom bodies [29]. *D***α***7* is also expressed during development. *D***α***7* transcripts are first detected in embryos 14–16 hr after egg laying, with expression in L1, L2, late L3, throughout pupation and in adult. Larva and adult expression are primarily detected in the central nervous system [30–33]. Expression at the mushroom body calyces has been confirmed by other groups [34–36], including expression at the calyces of larval mushroom bodies [35]. The mushroom bodies in turn have been implicated in ethanol reward, and ethanol-induced hyperactivity [37,38]. Previous studies on the effects of nicotine on *Drosophila* behavior have shown that acute nicotine exposure in flies disrupts their ability for negative geotaxis, a simple innate behavior in fruit flies [38–40], and that this effect is mediated by alpha-bungarotoxin-sensitive *Drosophila* nAChRs via efflux of biogenic amines [41]. A study by Ren et al. [42] implicated *D***α***7* in chronic nicotine-induced hyperactivity in *Drosophila*. The homology of *D***α***7*, its expression in regions of the fly nervous system associated to drug-reward, and its involvement in nicotine-induced behaviors in adult flies make *D***α***7* a candidate for mediating nicotine’s effects during developmental exposure.

The aim of this study was to establish a *Drosophila* model for the effects of developmental nicotine exposure on normal development and on adult behavior. The questions guiding this research were: 1) What are the effects of developmental nicotine exposure in *Drosophila melanogaster* development and behavior? 2) Are the effects of developmental nicotine exposure in *Drosophila melanogaster* similar to those described in other organisms? 3) Are these effects mediated by similar molecular mechanisms, such as nAChRs, and in particular via *D***α***7*? I hypothesized that developmental nicotine exposure would have similar effects in *Drosophila* to those documented in other organisms, and that these effects would be mediated by *D***α***7*. I found that, as in mammals, developmental exposure to nicotine decreased survival and adult weight. In addition, nicotine exposure delayed development, and decreased adult sensitivity to nicotine and ethanol. *D***α***7* mediated nicotine-induced effects on survival, developmental delay and may also have a role on nicotine-induced sensitivity to acute nicotine. This research also uncovered a role for *D***α***7* on acute ethanol sensitivity in *Drosophila*. The effects described for developmental nicotine exposure are concordant with what has been shown in humans and other model organisms, underlying the high conservation that regulates the deleterious effects of developmental nicotine exposure in humans and other mammals.

*Drosophila melanogaster* has been a great model system to identify novel genes and molecular mechanisms for drugs, and its development is well characterized. These two factors and the findings shown here make *Drosophila* a suitable model organism to uncover novel mechanisms underlying the developmental effects of nicotine.

## Materials and methods

### *Drosophila* strains and culture

Flies were reared in a light-dark controlled incubator on a 12h:12h light:dark cycle, kept at 25°C and 70% humidity on a standard cornmeal/molasses/yeast/agar medium. Experiments were carried out on flies of *w*^*1118*^Berlin (*w*B) genetic background from the Heberlein Lab at UCSF, except for the experiments with the *D***α***7* mutant. The *D***α***7* deletion line, *P***Δ***EY6* (EY6), and its precise excision control line, *P***Δ***EY5* (EY5), were a gift from Amir Fayyazuddin (Dart Neuroscience, San Diego, CA, USA), who characterized these lines [29]. These lines are of a mixed background onto which the wild-type *yellow* and *white* genes were reintroduced. Nicotine and ethanol sensitivity assays used 17–22 males aged 2–4 days after eclosion. Flies used on behavioral assays were subjected to brief (<5min) CO_2_ anesthesia during collection two days before the behavioral assay.

### Nicotine exposure

The developmental drug exposure protocol was adapted from McClure et al., [23]. Nicotine concentrations used were determined experimentally and are similar to what Ren et al. used for chronic nicotine exposure in adult flies [42]. Developmental exposure was defined as starting from the egg until 2 days after eclosion. Egg collections were taken for 3–4 hours on petri dishes containing 0, 0.1, 0.2, 0.3 or 0.4 mg/ml nicotine-laced food (Nicotine: N3876, Sigma-Aldrich) capping bottles placed inside a 25°C and 70% humidity incubator. The petri dishes were then collected from the bottles and placed back in the incubator overnight. The next day, newly hatched larvae (50–100 depending on the experiment) were transferred to vials containing either regular food or nicotine food and placed in a water bath with 1 inch of water to which a drop of hand soap had been added. The soap prevented mold from growing and the water bath created additional humidity and provided more consistent nicotine concentrations and results across experiments. To determine critical periods for the effects of nicotine, exposure was restricted to specific stages of development as follows: during egg maturation, for the Embryo stage (E); from 1^st^ to 3^rd^ instar larvae, for Larva stage (L); during metamorphosis, for the Pupa stage (P).

### Developmental assays

Flies were exposed to different concentrations of nicotine throughout development, as explained above. To assess survival and eclosion delay, the number of newly eclosed flies was counted between day 9 and 16 after egg laying (AEL) and the data was used to calculate percent survival and obtain cumulative eclosion rate plots, from which the eclosion time to 50% (ET50) was calculated. ET50 is defined as the time at which 50% of the total number of eclosed flies by day 16 after egg laying had eclosed. To determine changes in adult dry weight, flies were collected into eppendorf tubes 2 days after eclosion, desiccated for 9 days and weighed. To estimate % of eclosed pupa the total number of pupae and the number of eclosed pupa were counted at 16 days after egg laying and the % of eclosed pupa was calculated as: (# of eclosed pupa / total number of pupa)*100. Flies tested on behavioral assays were reared on nicotine starting one day before the flies reared on control food to compensate for the developmental delay of the nicotine exposure protocol.

### Nicotine sensitivity assay

A negative geotaxis assay was used to determine nicotine sensitivity [39]. Briefly, 2.5 **μ**l of 19 mM nicotine in 200 proof ethanol solution was pipetted onto an electric resistor (2 ohms, 1 watt) wrapped around stainless steal nails protruding through a rubber plug. The ethanol was allowed to evaporate for at least 2 minutes. Flies were transferred to a glass vial closed by the plug with the resistor side inside the vial. A power supply was used to pass a 30 second pulse of 2 mA current through the resistor, which heated the resistor and volatilized the nicotine. Flies were exposed to nicotine in this container for 1 minute and transferred to a graduated cylinder covered with a plastic mesh to facilitate climbing. After transfer, the number of flies on the bottom of the graduated cylinder was counted every minute for 4 minutes for experiments with the *w*B strain and for 5 minutes for experiments with the EY5 and EY6 strains. Those data were used to calculate percent impairment (inability to climb) over time. The order of testing for control and experimental flies was swapped every trial. There was variability in the nicotine exposure due to changes in the resistors as they were used. Grubbs’ test was used to exclude outliers.

### Ethanol sensitivity assay

The loss-of-righting-reflex (LORR) test for ethanol sensitivity was used to determine cross-tolerance between nicotine exposure and ethanol [40,43]. Flies were transferred to perforated tubes through which ethanol vapors were constantly flowing to an ethanol concentration of 100:50 (ethanol vapor: humidified air). The tubes were spun at 2.5 or 5 minute intervals and the number of sedated flies (flies that have lost the ability to right themselves) was counted at each time point. From these data the time to 50% sedation (ST50) was calculated.

### Nicotine concentration

To determine nicotine concentration larvae (at the end of the 3^rd^ instar) or adult flies (4 days after eclosion, and 2 days after the end of nicotine exposure) were frozen, homogenized in 50 mM Tris-HCl (pH 7.5*)* and the homogenate centrifuged at 14,000 r.p.m for 20 min at 4°C. The supernatants were analyzed by gas chromatography at the UCSF Tobacco Biomarkers Core to determine cotinine concentration, a nicotine metabolite. I chose this metabolite instead of nicotine because the half-life of nicotine is only 2–3 hours and measurements would be done 2 days after the ending of the nicotine exposure [44]. It has been shown that insects also metabolize nicotine to cotinine using similar detoxifying pathways as mammals [45,46].

### Ethanol absorption

Internal ethanol concentration was measured from whole-fly homogenates of flies reared on 0.0 or 0.3 mg/ml nicotine food after groups of 15–20 flies were exposed to ethanol vapor for 0, 5 or 15 minutes. Flies were then frozen on dry ice and stored at -80°C. Flies were homogenized in 50 mM Tris-HCl (pH 7.5*)* and the homogenate was centrifuged at 14,000 r.p.m for 20 min at 4°C. Ethanol concentrations in supernatants were measured using an alcohol dehydrogenase-based spectrophotometric assay (Ethanol Assay Kit REF 229–29, by Genzyme Diagnostics). To calculate fly internal concentration we first determined fly water content as the difference between wet weight and dry weight for larvae or flies reared on control or nicotine food.

### qPCR

Dissected head fractions from *w*Berlin 3^rd^ instar larvae or whole adult flies reared on control or nicotine food were snap frozen on dry ice and stored at -80°C. The heads of the frozen adult flies were then separated. Total RNA was extracted from either the larval head fractions or adult heads using TRIzol (Ambion). The mRNA in the total RNA was reversed transcribed using TaqMan Reverse Transcription Reagents (Applied Biosystems). The cDNA was analyzed by quantitative real-time PCR using the ABI PRISM 7700 Sequence Detection System (Applied Biosystems). The probe and primers for *D***α***7 (gfa*: Dm01799687_m1), and *rpl32* (Dm02151827_g1) were obtained from Applied Biosystems. The *rpl32* transcript levels were used as an endogenous normalization control for RNA samples and relative mRNA abundance was calculated using the comparative **ΔΔ**Ct method. Each experimental sample was run in triplicate, control samples were run in either triplicate or duplicate. Ct values with more than 1.0 difference within replicates were removed.

### Data analysis

Values shown are mean ± SEM. The number of samples, the number of independent experiments and the statistical test used for each data set are reported in the corresponding figure legends. Statistical comparisons were done on SPSS. In summary, statistically significant differences at a significance level set as p<0.05 were determined between control conditions (no nicotine or wild-type) and experimental conditions. Data was subjected to Levene’s test of equality of variance, and depending on the results data were analyzed with parametric or non-parametric tests. If variances were not significantly different, Student’s t-test, ANOVA followed by Dunnett’s post hoc test or a MANOVA repeated measures model followed by Dunnett’s test were used to determine differences between control and experimental conditions. If variances were significantly different, Mann-Whitney U test, or a Kruskal-Wallis test followed by Dunn-Bonferroni pairwise comparisons were used. Asterisks on graphs denote significance as follows, one for p<0.05, two for p<0.01, three for p<0.001. Sample size per experiment is reported as follows: n ≥ number of samples per condition from n ≥ number of independent experiments. “Sample” for each experiment is defined in the corresponding figure legend.

## Results

### Exposure to nicotine during development decreases survival, delays eclosion and reduces adult weight

Prenatal nicotine exposure increases morbidity and mortality rates, delays growth and decreases birth weight in humans and rodents [13,14]. To determine if nicotine affects *Drosophila* development I measured survival rate, eclosion delay and dry weight of flies reared on food supplemented with increasing concentrations of nicotine (see Materials and Methods).

**Fig 1:**
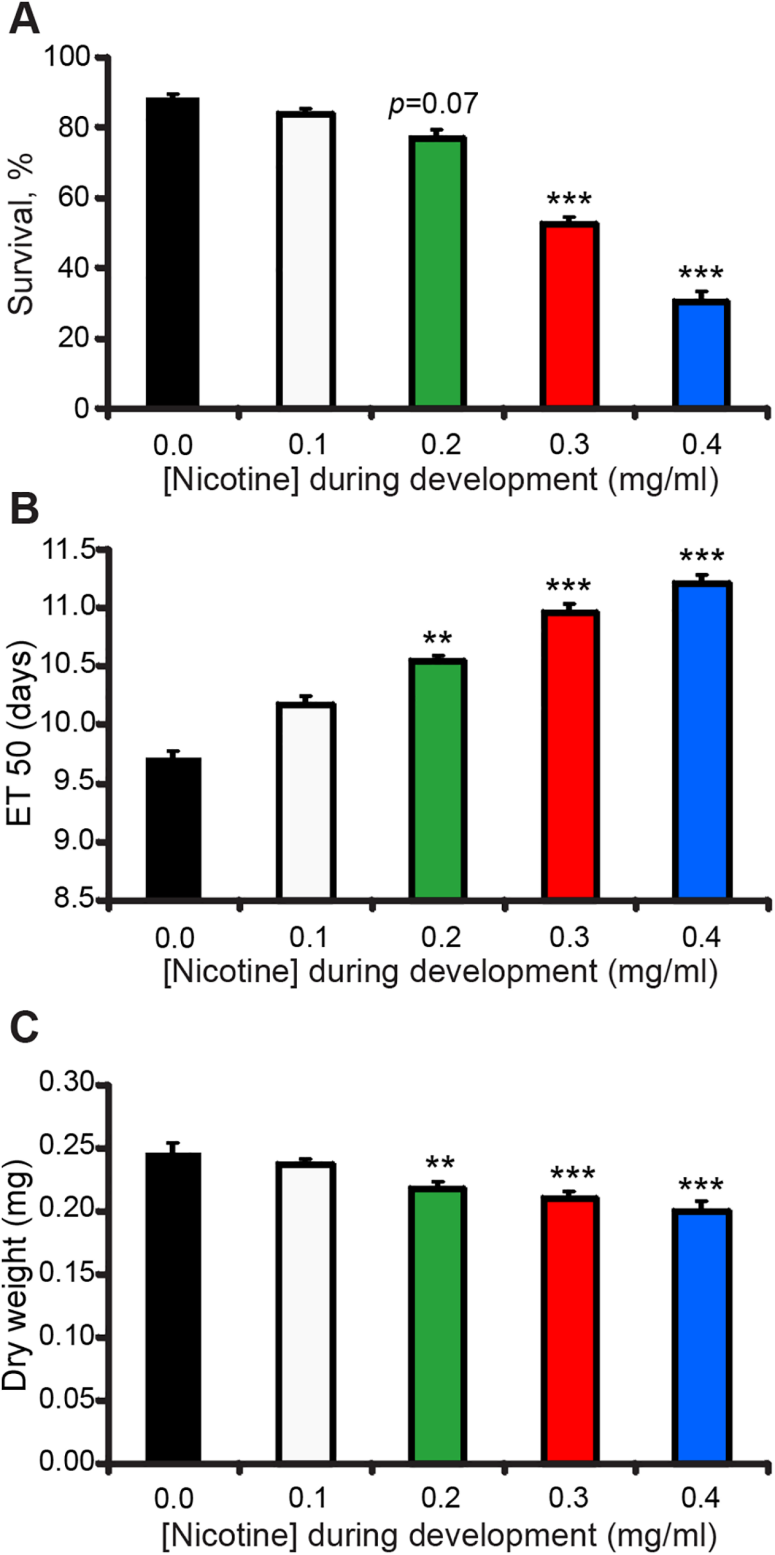
Nicotine-reared flies show reduced survival, developmental delay, and lower adult weight. Flies were reared on control or nicotine food from egg to adult and the number of eclosed flies over time was counted to estimate survival and developmental delay. For dry weight experiments, flies were collected 2 days after eclosion, desiccated for 9 days and weighed. (A) Percent survival by 16 days after egg-laying (ael) was significantly reduced from control (0.0 mg/ml nicotine) with increasing nicotine concentrations. (B) Time to 50% eclosion (ET50) was significantly longer than control with increasing nicotine concentrations. (A-B) Kruskal-Wallis followed by Dunn-Bonferroni pairwise comparison; only comparisons against control are shown; n ≥ 17 samples per nicotine concentration from n ≥ 4 independent experiments. Each sample is a fly vial with 50–100 animals exposed to nicotine. (C) Adult male dry weight was significantly lower than control with increasing nicotine concentrations. ANOVA followed by Dunnett's test; n ≥ 10 samples per nicotine concentration from n ≥ 4 independent experiments. Each sample is an eppendorf tube with ≥ 5 flies per tube to weigh.

First, I determined the survival rate of adult *Drosophila* from a known number of larvae feeding on nicotine food, picked from eggs laid on nicotine-laced collection dishes. As nicotine concentration increased, survival of larvae into adulthood sharply decreased (Fig 1A). Reduced survival was due to lethality during the larval stages, as the % of eclosed pupae, which represents the amount of eclosed pupae out of the total number of pupae was not significantly different for flies reared on nicotine food versus flies reared in control food (S1A Fig). Next, I examined if rearing flies on nicotine affects the time it takes to eclose into an adult. To measure eclosion time I determined the average time at which 50% of the flies had eclosed from cumulative eclosion plots (ET50, see Materials and Methods). The ET50 for flies reared on nicotine food was significantly higher compared to that of flies reared on control food. The higher the nicotine concentration, the longer the developmental delay (Fig 1B). For example, the 0.3 mg/ml nicotine concentration delayed eclosion by about a day, with an ET50 of 10.9 ± 0.1 days after egg-laying (AEL) compared to 9.7 ± 0.6 days AEL for control (Fig 1B). No attempt was made to compensate for the increased lethality on the high nicotine concentrations. Hence, it is possible that flies that survived the treatment are more resistant to nicotine and that the ET50 at the higher concentrations may be underestimated.

I also tested if rearing flies on nicotine had an effect on the weight of adult flies. I observed that developmental nicotine significantly decreased adult dry weight for both male (Fig 1C) and female flies (S1B Fig). Interestingly, water content was also decreased for flies exposed to nicotine (S1C Fig). Based on these results I chose a nicotine concentration of 0.3 mg/ml for all subsequent experiments, which yields about 50% survival (52 ± 2%), and approximately a 1-day developmental delay.

**Fig 2:**
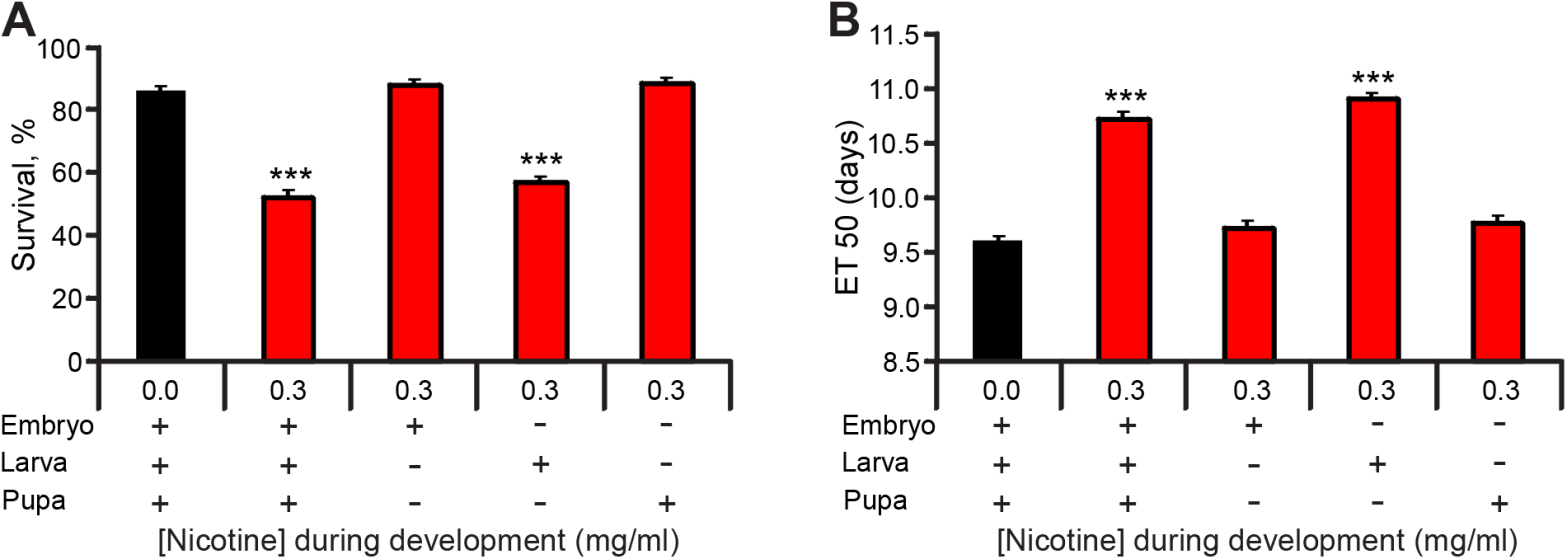
Nicotine exposure during the larval stage accounts for nicotine's effects on survival and developmental delay. Flies were reared on control or nicotine food during specific stages of development and the number of eclosed flies over time was counted to estimate survival and developmental delay. (A) Percent survival by 16 days ael was significantly reduced between control (black bar) and flies reared on nicotine food throughout development, or only during the larval stages. Nicotine exposure during embryogenesis or metamorphosis had no effect. (B) ET50 was significantly longer than control ET50 with nicotine exposure throughout development, or only during larval stages. Exposure during embryogenesis or metamorphosis did not have an effect on ET50. (A,B) Kruskal-Wallis followed by Dunn-Bonferroni pairwise comparison; only comparisons against control are shown; n ≥ 25 samples per condition from n ≥ 4 independent experiments. Each sample is a fly vial with 50–75 animals exposed to nicotine.

To refine at which developmental stage nicotine was having its effects, I modified the protocol to restrict nicotine exposure in developing flies to only either the embryonic, larval, or pupal stages (see Materials and Methods) and compared the effects to control (no nicotine) or flies exposed to nicotine throughout development. Focusing on survival and eclosion rates, I found that nicotine exposure only during the larval stages, a time when nicotine is consumed, accounted for both the decreased survival (Fig 2A) and delayed eclosion (Fig 2B). These results demonstrate that nicotine has a detrimental effect on fly survival and development as in mammals [13] and that *Drosophila* is particularly sensitive to the effects of nicotine during its larval stage.

### Exposure to nicotine during development decreases adult nicotine sensitivity

Flies display nicotine sensitivity: acute exposure of adult flies to volatilized nicotine impairs their ability to climb in a negative geotaxis assay [39,40]. I asked if nicotine exposure during development alters acute nicotine sensitivity of adult flies. Flies were reared on control or nicotine-laced food, collected two days after eclosion, and transferred to vials with control food (no nicotine) for two days prior to testing for nicotine sensitivity on a negative geotaxis assay.

I first determined the effectiveness of the nicotine exposure protocol by measuring the levels of the nicotine metabolite cotinine. Flies were reared on nicotine or control food and frozen as either 3^rd^ instar larvae or adult flies (at what would have been the behavioral testing day). As expected, 3^rd^ instar larvae that had been reared on nicotine contained higher cotinine levels compared to larvae not exposed to nicotine during development (S2A Fig). This concentration decreased to baseline in adult flies (S2A Fig). These results show that nicotine has been metabolized by the time of behavioral testing in adulthood.

**Fig 3:**
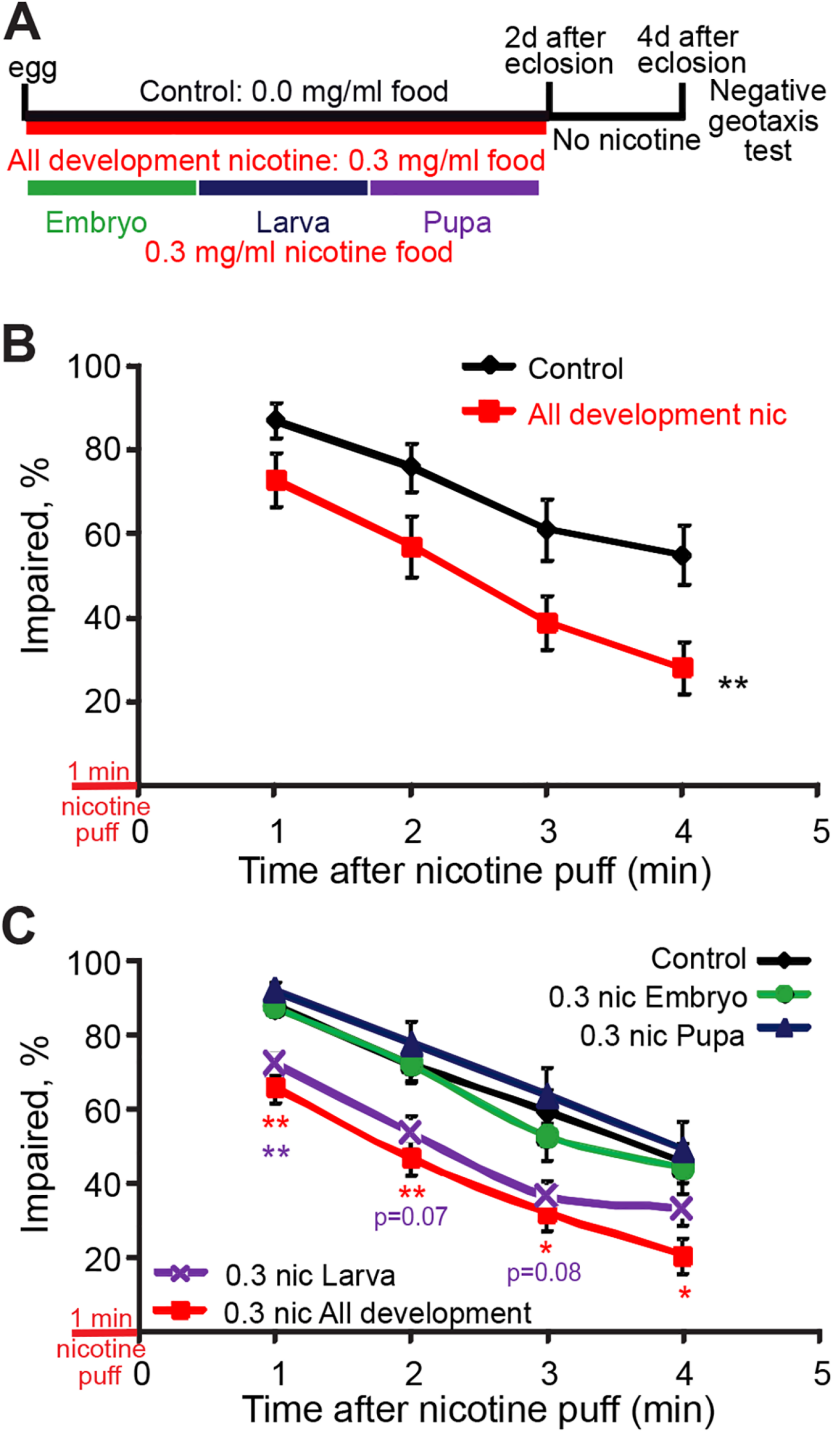
Nicotine-reared flies have decreased sensitivity to the acute effects of nicotine. Flies were reared on control or nicotine food during different developmental stages and males collected 2 days after eclosion and transferred to vials with control food. Flies were tested 2 days later on a negative geotaxis assay for nicotine sensitivity. (A) Developmental exposure protocols. (B) Flies reared on 0.3 mg/ml nicotine food (All development: red) displayed decreased sensitivity to the acute effects of nicotine on a negative geotaxis test compared to control flies reared on 0.0 mg/ml food. MANOVA repeated measures followed by Dunnett’s per time point, n ≥ 13 samples per condition from n = 3 independent experiments, each sample is a vial with 16–20 males). (C) Flies reared on 0.3 mg/ml nicotine food throughout development (All development: red line) or during the larval stages (Larva: purple line) showed decreased sensitivity to the effects of acute nicotine on a negative geotaxis test. Kruskal-Wallis by time point adjusted by Bonferroni correction to a p<0.0125 for significant differences, followed by Dunn-Bonferroni pairwise comparison; only comparisons against control are shown n ≥ 14 samples per condition from n ≥ 3 independent experiments, each sample is a vial with 15–20 males).

To test for adult nicotine sensitivity, I used the negative geotaxis test developed by Bainton et al. [39]. Briefly, flies received a puff of volatilized nicotine and transferred to a graduated cylinder, where measurements of the number of flies that remain at the bottom of the cylinder (% impaired) were taken every minute. These experiments were done either exposing flies throughout development or during specific developmental stages (Fig 3A). We observed that flies exposed to nicotine throughout development were significantly less sensitive to the impairing effects of acute nicotine exposure on this negative geotaxis assay when compared to flies reared in control food (Fig 3B).

Next I asked at which developmental stage nicotine acts to affect adult nicotine sensitivity. To do this, I compared adult responses to acute nicotine exposure in control flies with flies exposed to nicotine either throughout development or only during the embryonic, larval or pupal stages. Results showed that nicotine exposure during the larval stage was not significantly different to the effects of nicotine exposure throughout development, which resulted in resistance to acute nicotine. However, larval exposure alone had a milder effect, with significantly lower impairment only at 1min after acute nicotine exposure, and trends at 2min and 3min. I saw no statistically significant effect with exposure at the egg or pupal stages (Fig 3C).

Together these results show that developmental nicotine exposure throughout development reduces nicotine sensitivity as measured by impairment of negative geotaxis, while exposure at the larval stage contributes most saliently to this effect. Moreover, these effects are not due to residual nicotine at the time of behavioral testing, as levels of cotinine had decreased below detection at this time. This suggests that the effects could be due to alterations induced by nicotine exposure during development in pathways that normally determine nicotine sensitivity.

### Exposure to nicotine during development decreases adult ethanol sensitivity

Consumption of drugs of abuse by parents has been associated with increased consumption in offspring [47]. Maternal smoking has been associated with a higher incidence of alcohol abuse in the offspring [17]. The mechanisms for this are not well understood. Available clues come from studies looking at the relationship between ethanol sensitivity and ethanol consumption. Decreased ethanol sensitivity has been associated with increased incidence of alcohol abuse [48]. Moreover, decreased nicotine sensitivity (nicotine tolerance) has been shown to increase ethanol self-administration [49]. Animal studies have demonstrated the phenomenon of cross-tolerance between nicotine and ethanol, in which exposure to one drug decreases sensitivity to the other drug [50]. Therefore, I asked if developmental nicotine exposure leads to decreased ethanol sensitivity in adult flies. Flies were reared on nicotine as before, and collected two days before testing for ethanol sensitivity using the loss-of-righting-reflex (LORR) [40,43].

**Fig 4:**
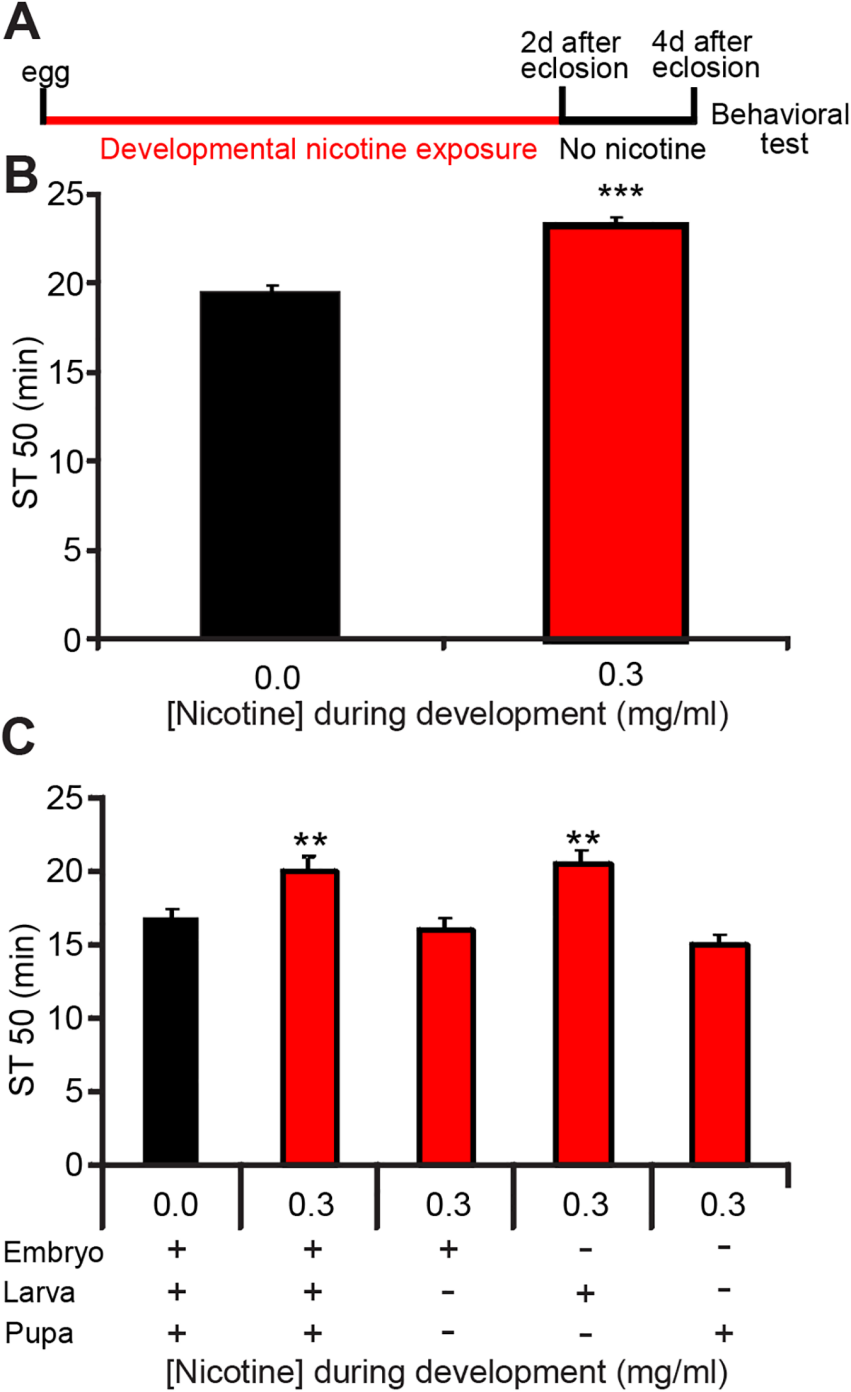
Nicotine-reared flies have decreased sensitivity to the sedative effects of ethanol. Flies were reared on control or nicotine food during different developmental stages and males collected 2 days after eclosion and transferred to vials with control food. Flies were tested 2 days later on the LORR assay for ethanol sensitivity. (A) Flies reared on 0.3 mg/ml nicotine food showed decreased sensitivity to the effects of acute ethanol exposure in the loss-of-righting-reflex (LORR) assay compared to control (0.0 mg/ml nicotine). (Student's t-Test, n ≥ 25 samples per condition from n = 6 independent experiments; each sample is a vial with 14–21 males). (B) Flies reared on 0.3 mg/ml nicotine food either during "All development" or during Larva displayed reduced sensitivity to the effects of acute ethanol exposure in the LORR assay compared to control. Nicotine exposure during Embryo or Pupa had no effect (ANOVA followed by Dunnett's, n ≥ 20 samples per condition from n ≥ 4 independent experiments; each sample is a vial with 16–21 males).

In the LORR assay, groups of flies are exposed to a continuous stream of ethanol vapor. The time it takes for 50% of the flies to display LORR, named ST50, is taken as a measure of their ethanol sensitivity. These experiments were done either exposing flies throughout development or during specific developmental stages (Fig 4A). I found that flies exposed to nicotine throughout development had lower sensitivity to ethanol-induced sedation (increased ST50) than control flies not exposed to nicotine during development (Fig 4B). Importantly, this difference was not due to ethanol absorption disparities, suggesting that developmental nicotine exposure did not alter ethanol pharmacokinetics (S2B Fig).

Next I asked at which developmental stage or stages nicotine acts to decrease ethanol sensitivity. Flies were exposed to nicotine at different developmental stages as before. Results showed that the larval stage was the only developmental stage where nicotine exposure decreased ethanol sensitivity. (Fig 4C).

These results indicate that exposure to nicotine during development decreases ethanol sensitivity. The data in Fig 3 shows that rearing flies on nicotine also decreases nicotine sensitivity. These results suggest that there might be common molecular and cellular mechanisms underlying the nicotine-induced changes in both, nicotine and ethanol sensitivity.

### *D*α*7* is involved in the effects of developmental nicotine exposure

Given the homology between the vertebrate *alpha7* and *D***α***7* subunit genes [28], and the involvement of *D***α***7* in chronic nicotine-induced hyperactivity [42], I tested if *D***α***7* had a direct role in the effects of developmental nicotine exposure described above. I used a strain of flies that contains a deletion of *D***α***7*, *P***Δ***EY6* (EY6), and the genetic background control line *P***Δ***EY5* (EY5) [29].

**Fig 5:**
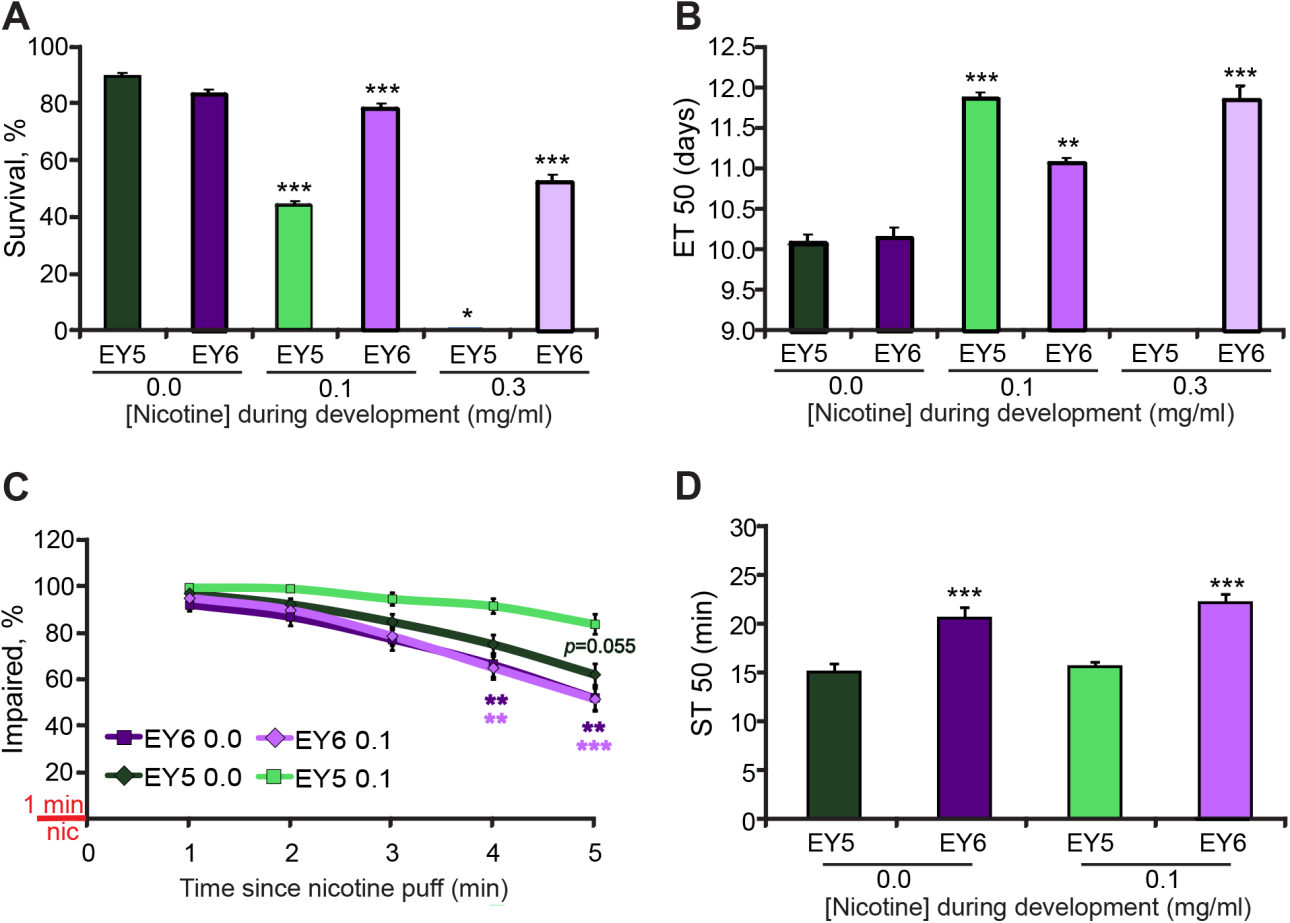
*D*α*7* mediates nicotine-induced mortality, developmental delay and acute nicotine and ethanol sensitivity. Flies were reared on control or nicotine food. Assays to determine mortality, developmental delay and drug sensitivity were carried out as before. (A) *D***α***7* mutant flies (EY6) had similar survival rate than their genetic control (EY5) when grown on normal food. However, EY6 flies survived more at higher nicotine concentrations (0.1, 0.3 mg/ml) than EY5. (B) EY5 and EY6 flies had a similar ET50 when reared on control food. However, a higher nicotine concentration (0.3 mg/ml) was needed to achieve a similar developmental delay to what was achieved with 0.1 mg/ml nicotine for EY5 flies. It was not possible to calculate ET50 for EY5 flies reared on 0.3 mg/ml nicotine because there were almost no survivors (A, B) Kruskal-Wallis followed by Dunn-Bonferroni pairwise comparison; only comparisons against control are shown, n ≥ 7 samples per condition from n ≥ 2 independent experiments; each sample is a fly vial with 60–100 animals exposed to nicotine. (C) EY5 flies reared on 0.1mg/ml nicotine food (light green line) had decreased sensitivity to the acute effects of nicotine on a negative geotaxis test. EY6 flies did not develop sensitivity to nicotine when reared in nicotine food. (Kruskal-Wallis by time point adjusted by Bonferroni correction to a p<0.01 for significant differences followed by Dunn-Bonferroni pairwise comparison between the 4 conditions–EY5 0.0, EY5 0.1, EY6 0.0 and EY6 0.1-; only comparisons against EY5 0.1 are shown, n ≥ 18 samples per condition from n = 6 independent experiments, each sample is a vial with 17–20 males). (D) EY6 flies reared on control food showed reduced sensitivity to the acute effects of ethanol on a LORR assay compared to the EY5 control flies reared on control food. Rearing EY5 or EY6 flies on 0.1 mg/ml nicotine food did not significantly change their acute ethanol response. Kruskal-Wallis followed by Dunn-Bonferroni pairwise comparison; only comparisons against the EY5 control are shown, n ≥ 18 samples per condition from n ≥ 3 independent experiments; each sample is a vial with 14–20 males.

First, to determine involvement of *D***α***7* on the developmental effects of nicotine, I exposed EY6 and EY5 flies to 0.0, 0.1 or 0.3 mg/ml nicotine from egg to 2 days after eclosion and determined percent survival and ET50. Almost none of the control (EY5) flies survived on 0.3 mg/ml. However, the *D***α***7* mutant line, EY6, was markedly resistant to nicotine’s effect on survival. EY6 flies reared on nicotine food survived at much higher rates than EY5 flies, reaching about 50% survival when reared on 0.3 mg/ml nicotine versus nearly 0% survival for EY5 flies at this concentration (Fig 5A). Next, I determined the effects of nicotine exposure on eclosion time for flies with the *D***α***7* mutation. The time to 50% eclosion (ET50) on control food was not significantly different between the EY5 and EY6 lines. However, EY6 flies were less affected by nicotine, having a shorter ET50 than EY5 flies at the 0.1 mg/ml concentration. At the 0.3 mg/ml concentration, only EY6 flies survived and displayed a developmental delay similar to that of EY5 flies at the 0.1 mg/ml nicotine concentration (Fig 5B). Based on these results, I used the 0.1 mg/ml concentration of nicotine for subsequent behavioral experiments.

Next, I tested nicotine sensitivity and ethanol sensitivity of EY5 and EY6 flies after developmental nicotine exposure. The proportion of impaired flies after acute nicotine exposure was similar during the first few minutes of the recovery, but differences emerged at 4 and 5 minutes after acute nicotine exposure (Fig 5C). Subsequent paired comparisons found that control EY5 flies reared on nicotine food (EY5 0.1) were more sensitive to the acute effects of nicotine than mutant EY6 flies reared in either control (EY6 0.0) or nicotine food (EY6 0.1), and that there was a trend towards a significant difference by 5 minutes when compared to EY5 flies reared on control food (EY5 0.0)(Fig 5C). There were no statistically significant differences in the response to acute nicotine between EY5 reared in control food and EY6 flies grown in either control food or nicotine food. These data suggest that EY5 flies developed sensitivity to the acute effects of nicotine on the negative geotaxis assay when reared in nicotine food. In contrast, EY6 flies reared in nicotine were not affected by the nicotine exposure during development, showing the same response to acute nicotine as EY6 flies reared in control food. EY6 and EY5 flies reared in control food had similar responses to acute nicotine. These results suggest that *D***α***7* is needed for the developmental-nicotine-induced sensitivity to acute nicotine seen in EY5 flies in this negative geotaxis assay. These results also suggest that *D***α***7* is not needed for acute nicotine sensitivity in this assay.

Then, I tested if the loss of *D***α***7* had any effects on ethanol sensitivity. These experiments showed that EY5 and EY6 flies have different sensitivity to ethanol. Mutant EY6 flies had reduced sensitivity to ethanol sedation relative to EY5 control flies, suggesting that *D***α***7* normally acts to promote ethanol sensitivity. However, developmental nicotine exposure did not affect the ethanol sensitivity observed in either EY5 or EY6 flies (Fig 5D). These results show that *D***α***7* regulates acute ethanol sensitivity.

There were discrepancies in the results we obtained with the *w*Berlin (*w*B) strain compared to those of the control EY5 strain. The EY5 strain had higher sensitivity to nicotine on survival and developmental delay, compared to wB. In addition, nicotine exposure during development in *w*B flies decreased nicotine sensitivity, while it increased nicotine sensitivity in EY5 flies. Developmental nicotine exposure increased ethanol sedation in *w*B, but had no effect in EY5 flies at the nicotine and ethanol concentrations tested. Studies have shown that genetic background can have an effect on behaviors such as locomotion, sleep, and sensitivity to anesthetics [51–53]. The contrasts we found are likely due to differences in genetic background. Future experiments knocking down *D***α***7* in flies of *w*B background would shed light on possible effects of genetic background on the role of *D***α***7* on specific effects of developmental nicotine exposure. The results may also have been affected by the high sensitivity to nicotine EY5 flies displayed, which made it impossible to test a higher nicotine concentration that may have affected EY6 flies more. It is possible that we may have seen significant differences in sensitivity for EY6 at higher nicotine concentrations.

**Fig 6:**
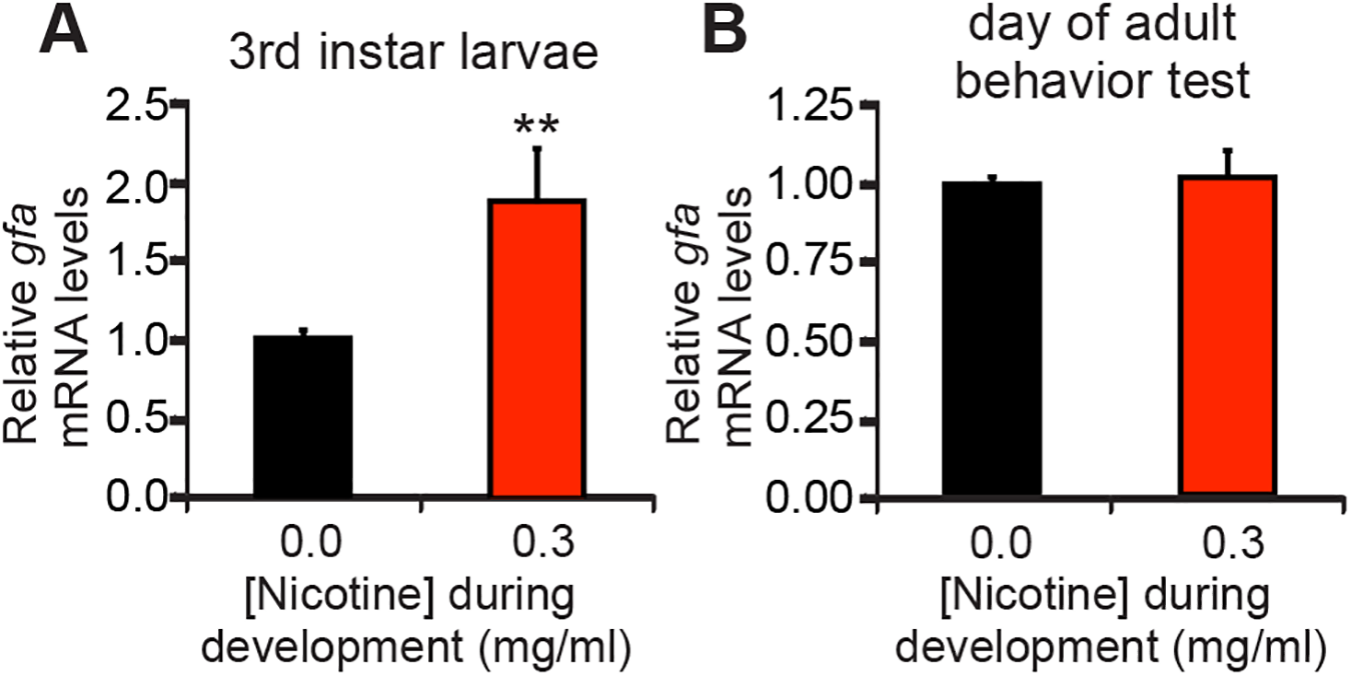
Developmental nicotine exposure modulates *gfa* transcript expression at the wandering 3^rd^ instar larval stage. Flies were reared on control or nicotine food and 3^rd^ instar larvae or adult flies were snap frozen. Head fractions from larvae or heads from adult flies were collected, RNA extracted, reversed transcribed and qPCR carried out. (A) *D***α***7* transcript expression was upregulated by developmental nicotine exposure measured at the end of the 3rd instar larva. (B) *D***α***7* expression was normal by the time of behavioral testing. (A,B) Mann-Whitney U Test, n = 3 samples per condition ran in triplicates from n≥ 2 independent experiments.

Last, I asked if developmental nicotine exposure was associated with transcriptional regulation of *D***α***7* mRNA. To do this, I determined expression levels of *D***α***7* transcripts by quantitative PCR in *w*Berlin flies reared in either control or nicotine food. Interestingly, developmental nicotine exposure from egg to the end of the 3^rd^ instar larvae increased *D***α***7* transcripts (Fig 6A). However, transcript levels for this gene at the time of behavioral testing (2 days after the last exposure to nicotine) were not statistically different (Fig 6B). These results show that *D***α***7* expression is acutely responsive to nicotine, with nicotine exposure during development leading to *D***α***7* transcript upregulation. Furthermore, transcript levels go back to normal if nicotine exposure ceases, which suggests that the effect of nicotine on adult behaviors is not likely due to transcriptional regulation of *D***α***7* by nicotine, but rather on neuroadaptations that nicotine induced on the nervous system during developmental exposure.

In summary, these results suggest that *D***α***7* mediates both, developmental and acute effects of nicotine. *D***α***7* mediates developmental-nicotine-induced lethality, and developmental delay; it may also mediate developmental-nicotine-induced acute nicotine sensitivity. *D***α***7* also promotes ethanol sensitivity.

## Discussion

### A *Drosophila* model to study the effects of nicotine exposure during development

I set out to establish a *Drosophila* model for the effects of developmental nicotine exposure on normal development and on adult behavior. To achieve this, I investigated the effects of nicotine exposure during development of the fruit fly to determine what those effects were and whether or not those effects were similar to those previously described. I found that, as in mammals including humans, developmental nicotine exposure affected survival, weight, and adult sensitivity to drugs. Specifically, I found that developmental nicotine exposure affected both development of the fly as well as adult sensitivity to nicotine and ethanol. Developmental effects included increased lethality, delayed development and decreased adult weight. The magnitude of these effects was concentration-dependent, with higher concentrations of nicotine achieving the strongest effects. Behavioral effects included decreased sensitivity to the effects of acute nicotine and decreased sensitivity to ethanol sedation. Nicotine exposure during the larval stages accounted for most of the effects of developmental nicotine exposure characterized. These effects were tested using a single dose for developmental nicotine exposure (0.3 mg/ml). If lower nicotine concentrations had been used, I hypothesize that the effects on behavior would be concentration-dependent. Future experiments could determine the minimum nicotine concentration needed to produce statistically significant effects on drug-sensitivity by adulthood. The effects on drug sensitivity were not likely due to nicotine’s direct action on targets in the fly’s nervous system during the assays, as time of testing was 2 days after the last nicotine exposure when levels of the nicotine metabolite cotinine had gone back to control levels. Therefore, the effects are likely due to nicotine action in the nervous system, such as effects on specific signaling pathways and/or components of the nervous system that determine normal sensitivity to nicotine and ethanol in adult flies.

Several of the effects I characterized for developmental nicotine exposure in *Drosophila* are similar to what has been described in other organisms including humans, rodents and zebrafish [13,14]. The effects on survival, developmental time and weight are similar to what McClure et al. reported for developmental ethanol exposure in *Drosophila* [23]. In zebrafish, developmental nicotine exposure decreases survival and growth, and alters startle and swimming behaviors [54,55]. Prenatal nicotine in rats has been shown to increase cocaine and methamphetamine self-administration [56,57]. These findings suggest that nicotine exposure during development has long-lasting effects on the brain’s reward system that predispose nicotine-exposed offspring to increased drug consumption later in life. My results also support the notion that nicotine exposure during development affects drug-induced behaviors in adulthood.

A difference between mammals and insects worth noting is that acetylcholine is the main neurotransmitter in the central nervous system in *Drosophila*, while glutamate is the main neurotransmitter in the central nervous system in mammals [58]. It is remarkable that despite this difference, there are similarities between the effects of developmental nicotine exposure in *Drosophila* characterized here and what has been described in mammals including humans. This suggests that glutamate in mammals and acetylcholine in insects could have a common target that mediates the effects of developmental nicotine exposure, possibly the reward system neural circuits and neurotransmitters. Dopamine and octopamine, a neurotransmitter closely related to noradrenaline, stand out as candidate neurotransmitters for such a role, as they act in the reward system of *Drosophila* [39,37,59,41]. Prenatal nicotine exposure has been associated to alterations in the dopaminergic system [16,60–62]. It would be interesting to determine if developmental nicotine exposure also affects development of the dopaminergic system in *Drosophila*.

The present results show that there is conservation in the effects of nicotine exposure during development between *Drosophila melanogaster* and other organisms, including mammals. This makes *Drosophila* a suitable model organism to study the effects of developmental nicotine exposure on the nervous system and behavior.

### Conservation of molecular mechanisms for nicotine’s actions

The last guiding question for this investigation was whether the effects of developmental nicotine exposure were mediated via nAChRs, in particular *D***α***7*. I chose to focus on *D***α***7* because it is expressed in regions of the fly brain associated to drug-reward, and its has been shown to mediate chronic nicotine exposure hyperactivity in adult flies, which suggested that it could also be involved in the effects of nicotine exposure during development [29,42]. I hypothesized that *D***α***7* might play a role in the effects of developmental nicotine exposure. I showed that *D***α***7* contributes to the developmental effects of nicotine on survival and developmental delay and it also mediates acute ethanol sensitivity. Further, I found that developmental nicotine exposure transiently increased expression of *D***α***7* transcripts (*gfa*) during the larval stage.

It is well established that nicotine is an agonist for nAChRs and thus can potentially act wherever acetylcholine normally acts on these receptors [8]. Studies with transgenic and mutant organisms are helping in understanding the role of different nAChRs on nicotine’s actions [9,63]. There is mounting evidence for nicotine-induced upregulation of nAChR receptors [64]. Neonatal nicotine exposure in rat elicits deficits in axogenesis and synaptogenesis and also changes in normal alpha7 expression patterns [65]. My results show a transient developmental upregulation of *D***α***7* mRNA: there was modulation of *D***α***7* mRNA levels during active nicotine exposure (development), yet the levels were normal by the time of behavioral testing (in adulthood). This suggests that the upregulation requires ongoing presence of nicotine, and presumably increased activation of nicotinic acetylcholine receptors, including D**α**7 receptors. Once nicotine is no longer present, the level of activation goes back to normal and the upregulation ends. Future experiments could determine if lower nicotine concentrations are able to upregulate *D***α***7* levels, I hypothesize that decreasing nicotine concentrations would lead to a proportional decrease in upregulation.

Next, it will be interesting to explore whether D**α**7 protein levels are modulated by developmental nicotine exposure, and to determine the timeline for upregulation of transcript to protein. It is possible that protein levels remain higher after transcripts have gone back to baseline. If that were the case, I cannot exclude contribution of left-over D**α**7 receptors in the behavioral effects of developmental nicotine exposure described. It would also be interesting to study the mechanism behind the developmental regulation of *D***α***7* transcripts. There may be post-transcriptional upregulation at play. For example, chronic nicotine has been shown to increase the number of **α**4**β**2 nAChRs in mammals via post-transcriptional mechanisms [9,64,66].

Because the developmental nicotine-induced effects on behavior were seen in adulthood, when *D***α***7* mRNA levels have returned to normal, I suggest that these effects were not due to acute activation of nAChRs by nicotine during the assays, but possibly to other changes nicotine exerted during development or more long-lasting effects on downstream effectors. The upregulation of *D***α***7* during the larval stage could interfere with normal development of the nervous system. The alpha7 receptor has an important role in mammalian nervous system development, including the transition of GABA from being an excitatory neurotransmitter to becoming an inhibitory neurotransmitter and glutamatergic synapse formation [10,67]. In addition, alpha7 activation has been shown to be deleterious in neonatal mice [68]. Here I have shown that *D***α***7* mediates developmental nicotine’s effects on survival and developmental delay. Moreover, the upregulation of *D***α***7* happened during the larval stage, and nicotine exposure during the larval stay was sufficient to decrease survival and extend development, this suggests that the upregulation in *D***α***7* transcripts could underlie these effects of developmental nicotine exposure. Future research in *Drosophila* could look further into the roles *D***α***7* has on nervous system development, in particular on neurotoxicity and synaptogenesis, and into the mechanisms that mediate nicotine’s effects on survival and developmental delay.

Bainton et al. showed that acute nicotine exposure of adult flies causes impairment in a negative geotaxis test, and that dopamine, a biogenic amine, mediates sensitivity to nicotine in this assay [39]. More recently, Zhang et al. showed that dopamine also mediates the chronic nicotine exposure-induced locomotor hyperactivity in adult flies described by Ren et al. [42,69]. In mammals and *Drosophila*, alpha7 receptors have been associated to biogenic amine release [41,63]. It will be interesting to test if dopamine mediates the developmental-nicotine-induced effect on acute nicotine sensitivity and whether nicotine exposure during development affects normal development of biogenic amine systems in *Drosophila*.

Repeated nicotine exposures in adult flies were reported to increase sensitivity to nicotine in the negative geotaxis assay via the cAMP/PKA pathway [70]. More recently, the *esg* gene and the microRNA cluster 310^c^ have been identified as having a role in acute nicotine sensitivity in adult flies [25]. These are additional pathways that could be involved in mediating the effects of developmental nicotine exposure.

Fuenzalida-Uribe et al. showed that **α**-bungarotoxin-sensitive nAChRs mediate the effects of nicotine on a similar negative geotaxis assay [41]. My results suggest a role for *D***α***7* in developmental-nicotine-induced sensitivity to acute nicotine, but the data does not support a role for *D***α***7* on acute nicotine sensitivity. It is possible that the **α**-bungarotoxin-sensitive nAChRs that mediate acute nicotine sensitivity are composed of other nAChRs subunits, which also bind **α**-bungarotoxin, such as the D**α**5 which has been shown to form functional homomeric channels in heterologous expression systems [71,72].

Ren et al. showed that *D***α***7* mediates chronic nicotine exposure-induced locomotor hyperactivity in adult fruit flies [42]. In mammals, alpha7 receptors have been implicated in nicotine withdrawal [73], and have also been shown to mediate long-term nicotine consumption [19]. Future research in *Drosophila* could test if *D***α***7* mediates nicotine withdrawal or long-term consumption.

There is evidence that ethanol interacts with nAChRs. At the molecular level, ethanol has been shown to inhibit the responses of alpha7 receptors [74]. At the behavioral level, nicotine has been shown to mitigate ethanol-induced ataxia and this effect is mediated by the alpha 7 receptors in the cerebellum [75,76]. D**α**7 is expressed in the mushroom bodies [29], which in the fly have a role in oviposition preference for alcohol, in mediating ethanol reward and in ethanol-induced hyperactivity [37,38,77]. This investigation revealed a novel role for *D***α***7* on ethanol sensitivity. It will be interesting to test if *D***α***7* knock down in the mushroom bodies is sufficient to modulate ethanol sensitivity.

## Supporting information

S1 Fig Additional effects of nicotine. Flies were reared on control or nicotine food from egg to adult and the number of eclosed pupae was counted over time. For dry weight experiments, flies were collected 2 days after eclosion, desiccated for 9 days and weighed. Fly water content was determined as the difference between wet weight at the time of collection and dry weight after desiccation for larvae or adult flies reared on control or nicotine food. (A) The percent of eclosed pupa was not affected by developmental nicotine exposure (Student's t-Test, n ≥ 50 samples per condition from n = 8 independent experiments. Each sample is a fly vial with 50–80 animals exposed to nicotine). (B) Adult female dry weight was significantly lower for flies reared on increasingly higher nicotine concentrations (Kruskal-Wallis followed by Dunn-Bonferroni pairwise comparison; only comparisons against control are shown; n ≥ 20 samples per nicotine concentration from n ≥ 4 independent experiments. Each sample is an eppendorf tubes with ≥ 5 flies per tube to weigh). (C) Water content was reduced in nicotine-reared flies compared to flies reared on control food, both at the 3rd instar larvae stage and as adults (larvae: Student's t-Test, n ≥ 7 samples per condition from n = 2 independent experiments, each sample is an eppendorf tube with ≥ 2 larvae to weigh; adult: Mann-Whitney-U test, n ≥ 10 samples per condition from n = 2 independent experiments, each sample is an eppendorf tube with ≥ 16 flies to weigh).
(TIF)

S2 Fig Developmental nicotine exposure increased cotinine concentration, but did not affect ethanol absorption. (A) Flies were reared on control or nicotine food (0.0 or 0.3 mg/ml nicotine, respectively, and their cotinine concentration was determined by gas chromatography at either the 3rd instar larva stage or the adult stage on the day that behavioral testing would have been performed. Adult flies had been removed from nicotine and were growing in normal food for 2 days at the time of collection for cotinine measurements. Developmental nicotine exposure generated a high internal cotinine concentration at the 3rd instar larval stage (p = 0.029) compared to flies reared on control food; cotinine level was not significantly different (p = 1) at the time of behavioral testing. Mann-Whitney U-test for comparing treatments at each developmental stage (n≥3 samples per condition per time point from 3 independent experiments, each sample is a homogenate from 50 adults or larvae reared in control or nicotine food). (B) Adult flies reared on 0.0 or 0.3 mg/ml nicotine were exposed to ethanol vapors for either 0, 5 or 15 minutes and their internal ethanol concentration was determined by a spectrophotometric assay. Developmental nicotine exposure did not affect ethanol absorption. Student's t-Test test comparing treatments at each time point showed no statistical differences between 0.0 and 0.3 mg/ml nicotine-exposed flies (n≥3 samples per condition per time point from 2 independent experiments, each sample is a homogenate from 15–20 male flies).
(TIF)
