# Supplementary figures and images for: A Drosophila model for developmental nicotine exposure
Source: PLoS One. 2017 May 12;12(5):e0177710. doi: 10.1371/journal.pone.0177710 (PMC5428972; doi:10.1371/journal.pone.0177710)

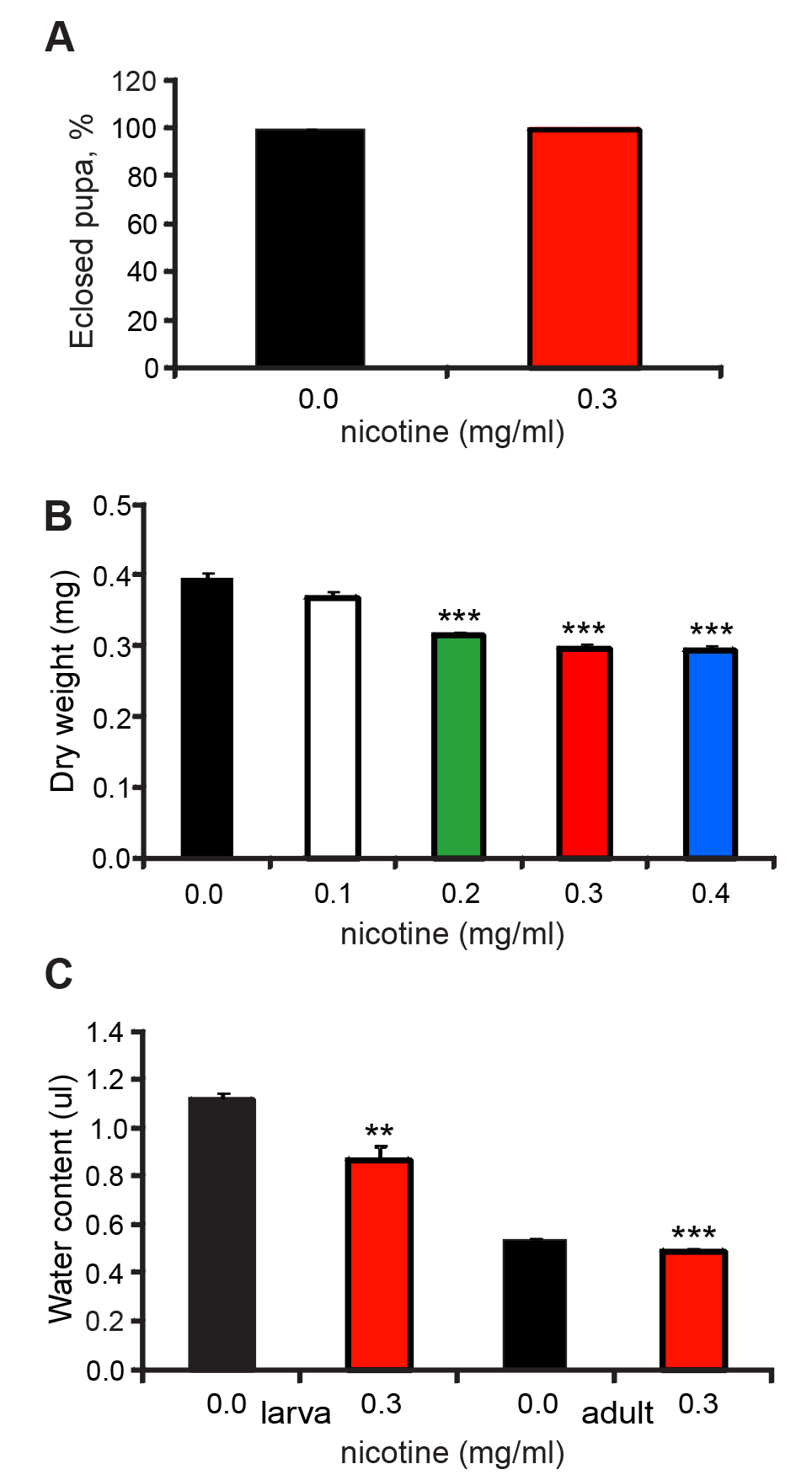

Supplement: S1 Fig — Flies were reared on control or nicotine food from egg to adult and the number of eclosed pupae was counted over time. For dry weight experiments, flies were collected 2 days after eclosion, desiccated for 9 days and weighed. Fly water content was determined as the difference between wet weight at the time of collection and dry weight after desiccation for larvae or adult flies reared on control or nicotine food. (A) The percent of eclosed pupa was not affected by developmental nicotine exposure (Student's t-Test, n ≥ 50 samples per condition from n = 8 independent experiments. Each sample is a fly vial with 50–80 animals exposed to nicotine). (B) Adult female dry weight was significantly lower for flies reared on increasingly higher nicotine concentrations (Kruskal-Wallis followed by Dunn-Bonferroni pairwise comparison; only comparisons against control are shown; n ≥ 20 samples per nicotine concentration from n ≥ 4 independent experiments. Each sample is an eppendorf tubes with ≥ 5 flies per tube to weigh). (C) Water content was reduced in nicotine-reared flies compared to flies reared on control food, both at the 3rd instar larvae stage and as adults (larvae: Student's t-Test, n ≥ 7 samples per condition from n = 2 independent experiments, each sample is an eppendorf tube with ≥ 2 larvae to weigh; adult: Mann-Whitney-U test, n ≥ 10 samples per condition from n = 2 independent experiments, each sample is an eppendorf tube with ≥ 16 flies to weigh). (TIF) [file pone.0177710.s001.tif]

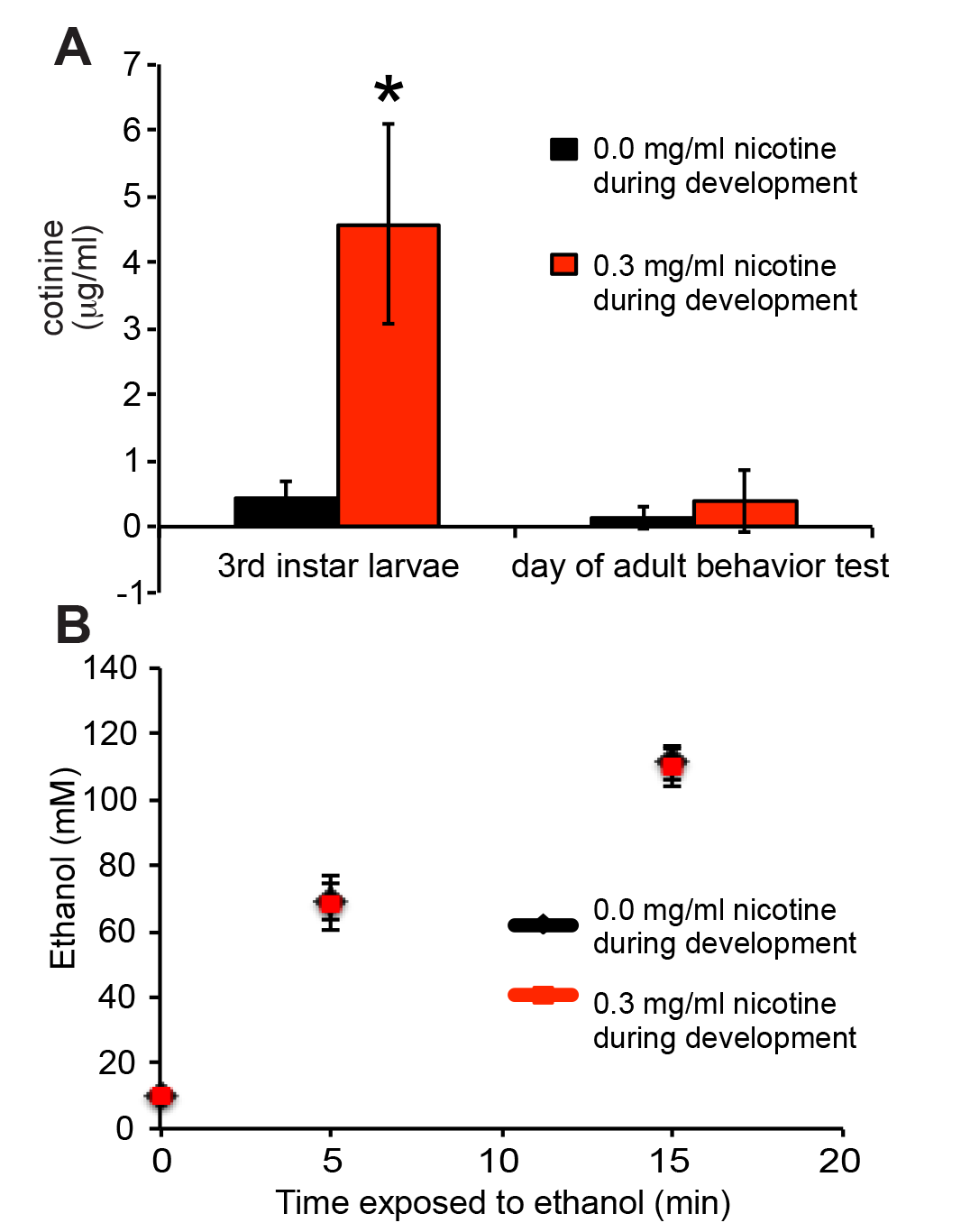

Supplement: S2 Fig — (A) Flies were reared on control or nicotine food (0.0 or 0.3 mg/ml nicotine, respectively, and their cotinine concentration was determined by gas chromatography at either the 3rd instar larva stage or the adult stage on the day that behavioral testing would have been performed. Adult flies had been removed from nicotine and were growing in normal food for 2 days at the time of collection for cotinine measurements. Developmental nicotine exposure generated a high internal cotinine concentration at the 3rd instar larval stage (p = 0.029) compared to flies reared on control food; cotinine level was not significantly different (p = 1) at the time of behavioral testing. Mann-Whitney U-test for comparing treatments at each developmental stage (n≥3 samples per condition per time point from 3 independent experiments, each sample is a homogenate from 50 adults or larvae reared in control or nicotine food). (B) Adult flies reared on 0.0 or 0.3 mg/ml nicotine were exposed to ethanol vapors for either 0, 5 or 15 minutes and their internal ethanol concentration was determined by a spectrophotometric assay. Developmental nicotine exposure did not affect ethanol absorption. Student's t-Test test comparing treatments at each time point showed no statistical differences between 0.0 and 0.3 mg/ml nicotine-exposed flies (n≥3 samples per condition per time point from 2 independent experiments, each sample is a homogenate from 15–20 male flies). (TIF) [file pone.0177710.s002.tif]
